# Editorial: Mental Healthcare: Pandemic and Beyond

**DOI:** 10.3390/medicina62020397

**Published:** 2026-02-19

**Authors:** Soumitra Das

**Affiliations:** 1 Department of Psychiatry, University of Melbourne, Melbourne, VIC 3010, Australia; soumitra.das@wh.org.au; 2 Department of Emergency Mental Health, Western Health, Melbourne, VIC 3021, Australia

Emergency mental health services became the backbone of healthcare delivery during the COVID-19 pandemic, particularly when most non-essential health services were curtailed or suspended. While this shift was both necessary and inevitable, the extraordinary burden placed on emergency mental health systems was evident in terms of workforce strain, resource limitations, and emotional exhaustion [1]. Yet, as often observed in periods of profound adversity, opportunities for psychological growth and adaptation can also emerge from sustained stress [2].

Within this Special Issue, Badura-Brzoza and colleagues explore the construct of ego-resiliency and its protective role in coping with generalised anxiety during pandemic-related stress. Their work highlights the importance of individual psychological resources in buffering against prolonged uncertainty and fear, offering valuable insights into resilience-based approaches for mental health professionals working in crisis contexts [3].

Equally important is a nuanced understanding of the factors contributing to anxiety, depression, and hopelessness among healthcare staff, as such understanding is central to designing meaningful and targeted support strategies. The contribution by Peñacoba-Puente et al. examines the roles of cognitive fusion and emotional exhaustion in the development of hopelessness, emphasizing how maladaptive cognitive processes and sustained emotional depletion may accelerate burnout. Their findings underscore the need for early identification and intervention strategies that address both cognitive and emotional dimensions of occupational stress [4].

To accurately capture the extent of psychological distress experienced during viral epidemics, the availability of valid and reliable assessment tools is essential. Sarhani-Robles and colleagues address this need by validating the Stress and Anxiety to Viral Epidemics-6 (SAVE-6) scale among medical students. Their study demonstrates good internal consistency and psychometric suitability, supporting the use of SAVE-6 as a brief and effective instrument to assess anxiety related to disease contagion among future healthcare professionals. Such tools are particularly valuable for both research and early preventive interventions [5].

The intersection between physical health and mental health is another critical theme explored in this Special Issue. Infectious illnesses present a bidirectional challenge, where physical vulnerability can exacerbate psychiatric morbidity and, conversely, mental health conditions and their treatments may increase the risk of physical complications. Miron’s et al. article draws attention to polypharmacy and the use of physical restraints as underestimated risk factors for pneumonia, prompting a re-examination of routine clinical practices. Complementing this, Zhao and colleagues highlight the association between clozapine treatment and pneumonia, reinforcing the importance of preventive strategies such as pneumococcal vaccination in high-risk psychiatric populations [6,7].

Finally, workforce wellbeing emerges as a central pillar of sustainable mental healthcare delivery. High workloads, insufficient psychological support, and persistent work–life balance stressors have been repeatedly identified as contributors to staff distress during and beyond the pandemic. Promoting mental health awareness, alongside the implementation of structured and systemic support plans, is therefore essential to maintain both workforce capacity and quality of care [8].

Collectively, this Special Issue on ‘Mental Healthcare: Pandemic and Beyond’ brings together eight articles that illuminate the complex interplay between psychological resilience, occupational stress, physical health, and systemic support. The contributions emphasise the necessity of addressing biopsychosocial factors to promote wellbeing, strengthen resilience, and ensure sustainable workforce development in mental health services during future public health crises and beyond.

## References

[1] McGuinness S.L., Johnson J., Eades O., Cameron P.A., Forbes A., Fisher J., Grantham K., Hodgson C., Hunter P., Kasza J. (2022). Mental Health Outcomes in Australian Healthcare and Aged-Care Workers during the Second Year of the COVID-19 Pandemic. Int. J. Environ. Res. Public Health.

[2] Bovero A., Balzani S., Tormen G., Malandrone F., Carletto S. (2023). Factors Associated with Post-Traumatic Growth during the COVID-19 Pandemic: A Systematic Review. J. Clin. Med..

[3] Badura-Brzoza K., Główczyński P., Dębski P., Błachut M., Skalski-Bednarz S.B. (2025). Ego-Resiliency and Coping Styles in Patients with Generalized Anxiety Disorder During the COVID-19 Pandemic. Medicina.

[4] Peñacoba-Puente C., Gil-Almagro F., García-Hedrera F.J., Carmona-Monge F.J. (2025). From Anxiety to Hopelessness: Examining Influential Psychological Processes Affecting Mental Health Status of Spanish Nurses During the COVID-19 Pandemic. Medicina.

[5] Sarhani-Robles A., Guillot-Valdés M., Lendínez-Rodríguez C., Robles-Bello M.A., Sánchez-Teruel D., Valencia Naranjo N. (2024). Psychometric Properties of the Anxiety Measure: Stress and Anxiety to Viral Epidemics-6 (SAVE-6) for Spanish Medical Students. Medicina.

[6] Miron A.-A., Ifteni P.I., Lungu A.-E., Dragomirescu E.-L., Dima L., Teodorescu A. (2025). Pre- and Post- COVID-19 Pandemic Pneumonia Rates in Hospitalized Schizophrenia Patients. Medicina.

[7] Zhao V., Gong Y., Thomas N., Das S. (2024). Clozapine and Pneumonia: Synthesizing the Link by Reviewing Existing Reports—A Systematic Review and Meta-Analysis. Medicina.

[8] Çerçizaj R., Kamberi F., Kiçaj E., Prifti V., Qirko S., Kokalla E., Rogozea L. (2025). Improving the Mental Health of Nursing Staff Seen from the Perspective of Staff a Preliminary Study. Medicina.

